# Rifampin pharmacokinetics in children, with and without human immunodeficiency virus infection, hospitalized for the management of severe forms of tuberculosis

**DOI:** 10.1186/1741-7015-7-19

**Published:** 2009-04-22

**Authors:** Hendrik Simon Schaaf, Marianne Willemse, Karien Cilliers, Demetre Labadarios, Johannes Stephanus Maritz, Gregory D Hussey, Helen McIlleron, Peter Smith, Peter Roderick Donald

**Affiliations:** 1 Department of Paediatrics and Child Health, Faculty of Health Sciences, Stellenbosch University and Tygerberg Children's Hospital, PO Box 19063, Tygerberg 7505, South Africa; 2 Department of Human Nutrition, Faculty of Health Sciences, Stellenbosch University, Tygerberg 7505, South Africa; 3 Division of Molecular Biology and Human Genetics and the Medical Research Council Centre for Molecular and Cellular Biology, Department of Science and Technology/National Research Foundation Centre of Excellence for Biomedical Tuberculosis Research, Faculty of Health Sciences, Stellenbosch University, PO Box 19063, Tygerberg 7505, South Africa; 4 Institute of Infectious Diseases and Molecular Medicine, University of Cape Town, Rondebosch 7701, South Africa; 5 Division of Clinical Pharmacology, University of Cape Town, Rondebosch 7701, South Africa; 6 Knowledge Systems, Human Sciences Research Council, Private Bag X9182, Cape Town 8000, South Africa

## Abstract

**Background:**

Rifampin is a key drug in antituberculosis chemotherapy because it rapidly kills the majority of bacilli in tuberculosis lesions, prevents relapse and thus enables 6-month short-course chemotherapy. Little is known about the pharmacokinetics of rifampin in children. The objective of this study was to evaluate the pharmacokinetics of rifampin in children with tuberculosis, both human immunodeficiency virus type-1-infected and human immunodeficiency virus-uninfected.

**Methods:**

Fifty-four children, 21 human immunodeficiency virus-infected and 33 human immunodeficiency virus-uninfected, mean ages 3.73 and 4.05 years (*P *= 0.68), respectively, admitted to a tuberculosis hospital in Cape Town, South Africa with severe forms of tuberculosis were studied approximately 1 month and 4 months after commencing antituberculosis treatment. Blood specimens for analysis were drawn in the morning, 45 minutes, 1.5, 3.0, 4.0 and 6.0 hours after dosing. Rifampin concentrations were determined by liquid chromatography tandem mass spectrometry. For two sample comparisons of means, the Welch version of the t-test was used; associations between variables were examined by Pearson correlation and by multiple linear regression.

**Results:**

The children received a mean rifampin dosage of 9.61 mg/kg (6.47 to 15.58) body weight at 1 month and 9.63 mg/kg (4.63 to 17.8) at 4 months after commencing treatment administered as part of a fixed-dose formulation designed for paediatric use. The mean rifampin area under the curve 0 to 6 hours after dosing was 14.9 and 18.1 μg/hour/ml (*P *= 0.25) 1 month after starting treatment in human immunodeficiency virus-infected and human immunodeficiency virus-uninfected children, respectively, and 16.52 and 17.94 μg/hour/ml (*P *= 0.59) after 4 months of treatment. The mean calculated 2-hour rifampin concentrations in these human immunodeficiency virus-infected and human immunodeficiency virus-uninfected children were 3.9 and 4.8 μg/ml (*P *= 0.20) at 1 month after the start of treatment and 4.0 and 4.6 μg/ml (*P *= 0.33) after 4 months of treatment. These values are considerably less than the suggested lower limit for 2-hour rifampin concentrations in adults of 8.0 μg/ml and even 4 μg/ml

**Conclusion:**

Both human immunodeficiency virus-infected and human immunodeficiency virus-uninfected children with tuberculosis have very low rifampin serum concentrations after receiving standard rifampin dosages similar to those used in adults. Pharmacokinetic studies of higher dosages of rifampin are urgently needed in children to assist in placing the dosage of rifampin used in childhood on a more scientific foundation.

## Background

Rifampin (RMP) is a key drug in modern antituberculosis chemotherapy by virtue of its unique ability to rapidly kill the majority of bacilli in tuberculosis lesions, prevent relapse and thus enable 6-month short-course chemotherapy. Inadequate absorption of RMP has been documented under different circumstances in a number of studies of adult tuberculosis patients; it has been found in human immunodeficiency virus (HIV) type-1-uninfected patients [1,2], amongst HIV-infected patients [3-5], and in some studies amongst both HIV-infected and HIV-uninfected patients [6-8]. In another recent study, although low RMP plasma concentrations were found in both HIV-infected and HIV-uninfected patients, HIV-infected patients were at greater risk for poor RMP absorption [9]. Others evaluating smaller numbers of patients have found adequate absorption amongst HIV-infected patients [10,11]. It has also been remarked that there is considerable intra-individual variation in RMP pharmacokinetics.

There is very little available information describing RMP pharmacokinetics in children and there is no information regarding the possible influence of HIV infection on RMP pharmacokinetics in children. This paper describes the pharmacokinetics of RMP in a group of children from the Western Cape Province of South Africa, both HIV-infected and HIV-uninfected, admitted to a tuberculosis referral hospital with severe forms of childhood tuberculosis.

## Methods

### Setting

The Brooklyn Hospital for Chest Diseases (BHCD) is a referral hospital for severe forms of tuberculosis occurring within the city of Cape Town. During the period January 2004 to December 2006 children aged 3 months to 13 years admitted to BHCD were eligible for enrolment in a study of the pharmacokinetics of antituberculosis agents. Admission to BHCD occurred approximately 1 month after commencement of treatment at the referring hospital.

### Diagnosis of tuberculosis

The diagnosis of tuberculosis was confirmed by culture of gastric aspirate, or sputum in older children, together with culture of cerebrospinal fluid in suspected cases of tuberculous meningitis; these investigations were usually carried out at referring hospitals before admission to BHCD. Mantoux testing was with tuberculin RT23 and induration was measured in the transverse diameter of the forearm after 48 to 72 hours. Induration of at least 10 mm was taken as indicative of *Mycobacterium tuberculosis *infection in HIV-uninfected children, but at least 5 mm in those HIV-infected. All parents or caregivers were questioned as to the presence in the child's household within the last year of cases of sputum-microscopy, smear-positive pulmonary tuberculosis. Chest radiographs were considered indicative of pulmonary tuberculosis in the presence of unequivocal mediastinal adenopathy; a diagnosis of tuberculous meningitis was accepted in the presence of hydrocephalus and basal enhancement seen on cranial computerised tomography, accompanied by appropriate cerebrospinal fluid changes. Two or more of the above clinical criteria were required to establish a diagnosis of probable tuberculosis.

### Treatment

Treatment for all forms of tuberculosis was with fixed-dose combinations (FDC) formulated for paediatric use; each tablet used during the intensive treatment phase contained RMP 60 mg, isoniazid (INH) 30 mg and pyrazinamide 150 mg (Rimcure^R^) and during the continuation phase RMP 60 mg and INH 30 mg (Rimactazid^R^). One tablet was used for every 5 kg increase in body weight. Rimcure^R ^and Rimactazid^R ^were supplied by Sandoz SA Pty Ltd Spartan, South Africa; these formulations are approved by the South African Medicines Control Council and were dispensed by the BHCD pharmacy. During the intensive phase ethambutol was added when the use of four drugs was considered advisable. Tuberculous meningitis was managed with the same FDC, but with ethionamide added, all given for 6 months. All tuberculosis treatment was given daily and observed by hospital nursing staff. On the day of a pharmacokinetic study, drug administration was undertaken by study personnel. All of the children were supplemented with a multivitamin syrup. This supplied pyridoxine 0.5 mg for children aged 0 to 4 years, but 1 mg for those >5 years; each 5 ml contained vitamin A 2300 IU, vitamin D 200 IU, vitamin B1 1 mg, vitamin B2 1.2 mg, nicotinamide 5 mg, vitamin C 35 mg and vitamin B12 0.0025 mg. All HIV-infected children received trimethoprim-sulphamethoxazole. Antiretroviral treatment consisted of two nucleoside reverse transcriptase inhibitors and ritonavir in children less than 3 years of age (Kaletra^R ^was not yet available), and ritonavir was replaced by efavirenz in children older than 3 years.

### Pharmacokinetic study

RMP plasma concentrations were measured within a week of admission to BHCD (approximately 1 month after initial admission to the referral hospital) and again 4 months after commencing treatment to evaluate the possible influence of nutrition, disease state and intra-individual variation on RMP pharmacokinetics. The children were nil per mouth from midnight and an intravenous catheter was inserted after the application of a local anaesthetic jelly (Ametop^R^). Medication was administered by study personnel. Blood specimens were taken at 45 minutes, 1.5, 3.0, 4.0 and 6.0 hours after dosing, immediately placed on ice and centrifuged within 30 minutes. A plasma sample of 1 ml was stored in polypropylene tubes at -80°C and protected from light until analysed. The specimens were analysed for RMP by liquid chromatography tandem mass spectrometry as described previously [12]; the limit of quantification was 0.1 μg/ml. The reference standard for RMP was purchased from Sigma (StLouis, MO, USA).

### Nutritional and clinical evaluation

Body mass and height, or length in those less than 2 years of age, and mid-upper arm circumference were measured by standard anthropometric methods by a qualified dietician and the mean of three readings taken [13,14]. Serum bilirubin and alanine transferase (ALT) were determined at the time of each pharmacokinetic evaluation.

The presence of abdominal nodes was evaluated by abdominal ultrasound at enrolment in all children.

Plasma C-reactive protein (CRP) was determined by an immunoradiometric assay at 1 and 4 months of treatment in all children [15].

The children's HIV status was determined by enzyme-linked immunosorbent assay and positive results confirmed by a second assay, or by polymerase chain reaction in children younger than 18 months. Counselling of the parents or legal guardians preceded HIV-testing and written informed consent was obtained.

### Statistics

For two sample comparisons of means the Welch version of the t-test was used; associations between variables were examined by Pearson correlation and by multiple linear regression. *C*_max _is the observed maximum value for each individual and *T*_max _the time at which *C*_max _was recorded. The area under the curve (AUC) over the period 0 to 6 hours after dosing was calculated by the linear trapezoidal rule. Experience with published reference ranges for RMP plasma concentrations from studies in healthy volunteers and tuberculosis patients have suggested that 2-hour concentrations of less than 8 μg/ml should be regarded as low and values less than 4 μg/ml as very low [5,8,16]. The 2 hours after dosing RMP plasma concentrations were thus calculated as [(2/3)(1.5 hour value+(1/3)(3.0 hour value)]. These calculated values were compared with interpolated values obtained from fitting a two-compartment regression model to the data. The correlation coefficients between the two sets of interpolated values were 0.983 and 0.982 at enrolment and at 4 months after treatment commencement, respectively, with negligible bias between the mean values.

The study was approved by the Institutional Review Board of the Faculty of Health Sciences of Stellenbosch University (No. 2003/054/N). All parents or legal guardians gave written informed consent for their children's participation in the study.

## Results

Sixty children, 26 HIV-infected and 34 HIV-uninfected, were enrolled in the study. Four children (all HIV-infected) were transferred back to referring hospitals shortly after enrolment due to complications that could not be managed at BHCD and were not studied further; two children (one each HIV-infected and HIV-uninfected) were discharged from hospital after completion of the first pharmacokinetic study and their data were not included in this analysis. Due to our inability to maintain the intravenous line, single data points were not available for 14 children (usually the 6-hour data point), two data points were missing from two children and three data points from one patient. Pharmacokinetic data were thus available for analysis on enrolment and after 4 months of treatment from 21 HIV-infected children and 33 HIV-uninfected children. The period between the start of treatment and admission to BHCD and enrolment in the study at BHCD did not differ between the HIV-infected and HIV-uninfected children: 34.2 days (standard deviation (SD) 21. 8) and 38.0 days (SD 19.3), respectively (*P *= 0.49).

Two HIV-infected children were already receiving antiretroviral treatment at the time of admission to BHCD and a further seven children were commenced on antiretroviral treatment, but this occurred only shortly before the completion of the second pharmacokinetic study.

Demographic, diagnostic and clinical features of the children are summarised in Table 1. Overall a culture of *M. tuberculosis *was obtained from 28 (51%) children and in a further three HIV-infected children with negative cultures, acid-fast bacilli were seen on microscopy of gastric aspirates. Only four (20%) HIV-infected children had a positive Mantoux test and this was greater than 10 mm in each case. A majority of children suffered from one or other form of malnutrition; although the proportion of HIV-uninfected children suffering from kwashiorkor and with a body mass less than the third percentile for age was similar to that amongst HIV-infected children, significantly more HIV-infected children were marasmic.

**Table 1:** Demographic, diagnostic, clinical and radiological features of human immunodeficiency virus (HIV)-infected and HIV-uninfected children being treated for tuberculosis.

	**Human immunodeficiency virus-infected** ***N *= 21**	**Human immunodeficiency virus-uninfected** ***N *= 33**	***P *value**
Age (years)	3.73	4.05	0.68
Male sex	12 (57%)	16 (48%)	0.58
Culture of *M. tuberculosis *or acid-fast bacillus smear seen on microscopy^1^	10 (48%)	18 (60%)	0.16
Household tuberculosis contact	14 (67%)	23 (70%)	0.82
			
			
Mantoux test^2^			
≥ 10 mm	4 (20%)	30 (91%)	<0.001
≥ 5 mm	-	-	
			
Clinical features			
			
Pulmonary tuberculosis	18 (86%)	27 (82%)	0.72
Tuberculous meningitis	7 (33%)	19 (58%)	0.16
			
Nutritional status			
			
Mass <3rd percentile for age	8 (38%)	13 (39%)	>0.99
Kwashiorkor	6 (29%)	6 (18%)	0.50
Marasmus	8 (38%)	3 (9%)	0.014
Marasmic kwashiorkor	4 (19%)	3 (9%)	0.41
			
Radiological features			
			
Hilar adenopathy	11 (52%)	18 (55%)	>0.99
Lobar opacification	9 (43%)	10 (30%)	0.39
Micronodular opacification	5 (24%)	7 (21%)	>0.99
Cavitation	0	6 (18%)	0.072
Abdominal nodes^3^	5 (24%)	6 (18%)	0.73

^1^Culture and microscopy not carried out in three human immunodeficiency virus (HIV)-uninfected children.

^2^Mantoux test not read in one HIV-uninfected child.

^3^Abdominal nodes visualised on ultrasound.

Table 2 summarises certain anthropometric features and CRP concentrations of the children on enrolment (after 1 month of treatment) and after 4 months treatment. At neither assessment did the anthropometric findings differ between the HIV-infected or HIV-uninfected children. At the 4-month assessment the anthropometric parameters showed improvement in both groups of children. On enrolment the mean CRP concentrations were raised in both groups of children, but were significantly higher in the HIV-infected children; by the 4-month assessment the CRP concentrations had declined in both groups, but while those of the HIV-uninfected children had normalised, with the exception of two children, those of the HIV-infected children remained elevated. With regard to liver function evaluation, no child had a raised serum bilirubin; at 1 month after commencement of treatment serum ALT was raised (more than 37 U/litre) in three (14.3%) HIV-infected children and was more than three times the normal level in two children (9.5%), while amongst the HIV-uninfected children, four (12.1%) children had an increased ALT and this was more than three times the normal level in two (6.1%) At 4 months ALT was increased in 6 (28.6%) HIV-infected children, but more than three times the normal in only two (9.5%) and amongst the HIV-uninfected ALT was raised in four children (12.1%) and in none was the value more than three times the normal level.

**Table 2:** Anthropometrical evaluation and serum C-reactive protein levels in human immunodeficiency virus (HIV)-infected and HIV-uninfected children being treated for tuberculosis on enrolment after 1 month and 4 months of treatment.

	**Human immunodeficiency virus-infected** ***N *= 21 (SD)**	**Human immunodeficiency virus-** **uninfected** ***N *= 33** **(SD)**	***P *value**	**Human immunodeficiency virus-infected** ***N *= 21** **(SD)**	**Human immunodeficiency virus-uninfected** ***N *= 33** **(SD)**	***P *value**
	On enrolment			After 4 months of treatment		
				
Weight (kg)	12.26 (5.18)	13.97 (6.94)	0.30	13.06 (5.33)	15.04 (8.01)	0.29
Length/height (cm)	86.80 (16.22)	91.53 (20.85)	0.36	89.26 (16.39)	93.67 (20.37)	0.40
Body mass index	15.59 (1.62)	15.71 (1.91)	0.80	15.74 (1.53)	16.05 (1.71)	0.51
Mid-upper arm circumference (cm)	14.28 (2.16)	15.07 (2.39)	0.21	14.44 (2.22)	15.46 (2.63)	0.16
Serum C-reactive protein (mg/litre)	39.97 (54.55)	13.42 (17.96)	0.06	22.47 (22.28)	4.15 (4.96)	0.002

SD, standard deviation.

The children received a mean RMP dosage of 9.61 mg/kg (range 6.47 to 15.58, SD 1.69) for the pharmacokinetic study on enrolment and 9.63 mg/kg (range 4.63 to 17.8, SD 2.26) during the 4-month study (*P *= 0.93). On enrolment one HIV-uninfected child received a RMP dose of less than 8 mg/kg and two HIV-uninfected children and one HIV-infected child a dose of more than 12 mg/kg; during the 4-month assessment two HIV-uninfected children received a dose of less than 8 mg/kg and three HIV-uninfected children a dose of more than 12 mg/kg.

**Figure 1:**
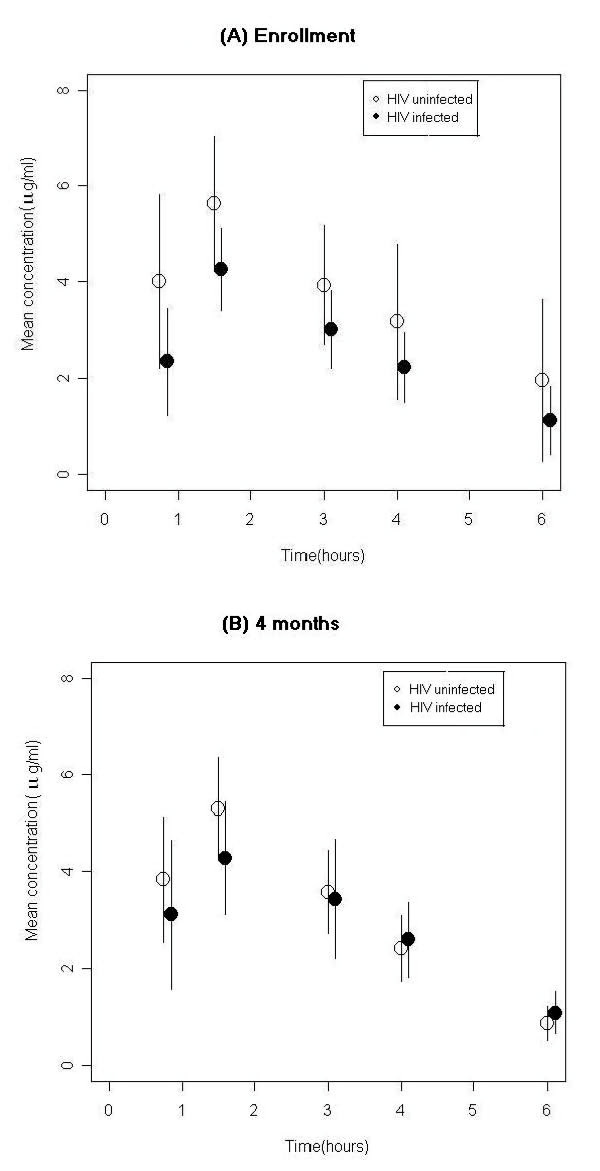
**Rifampin plasma concentrations (μg/ml) in human immunodeficiency virus (HIV)-infected and HIV-uninfected children being treated for tuberculosis determined on enrolment (A) and after 4 months of treatment (B)**.

Table 3 gives the RMP plasma concentrations determined at 45 minutes, 1.5 hours, 3.0 hours, 4.0 hours and 6.0 hours and those calculated for 2 hours after dosing 1 month and 4 months after commencing treatment; Figure 1 illustrates these serum concentrations in HIV-infected and HIV-uninfected children. The mean AUC_0–6 _on enrolment was 14.88 and 18.07 μg/hour/ml (*P *= 0.25) in HIV-infected and HIV-uninfected children, respectively, and after 4 months of treatment 16.52 and 17.94 μg/hour/ml (*P *= 0.59). While a majority of children have a consistent AUC_0–6 _on both occasions there was considerable inter-individual and intra-individual variation evident. Thus, the AUC_0–6 _of all 55 children was 16.81 (SD 10.82) on enrolment and 17.39 (SD 9.74) after 4 months of treatment, but the correlation of the AUC_0–6 _on enrolment with that after 4 months of treatment was low (*r *= 0.313, *P *= 0.022). There was no correlation between AUC_0–6 _and body mass index either at enrolment (one outlier excluded) or at 4 months of treatment (*r *= 0.109, *P *= 0.444; *r *= 0.014, *P *= 0.920, respectively).

**Table 3:** Mean rifampin plasma concentrations (μg/ml) in human immunodeficiency virus (HIV)-infected and HIV-uninfected children being treated for tuberculosis on enrolment after 1-month and 4-months of treatment.

**Time after dosing (hours)**	**Enrolment (SD)**					**4 months (SD)**				
	*N*	Human immunodeficiency virus-infected	*N*	Human immunodeficiency virus-uninfected	*P* value	*N*	Human immunodeficiency virus-infected	*N*	HIV-uninfected	*P *value

0.75	21	2.35(2.54)	32	4.02(5.10)	0.12	20	3.11(3.45)	33	3.84(3.72)	0.47
1.5	21	4.26(1.96)	33	5.63(4.07)	0.10	21	4.28(2.66)	33	5.31(3.01)	0.19
2.0^1^	21	3.90(1.67)	33	5.07(3.59)	0.11	21	4.00(2.04)	32	4.61(2.43)	0.33
3.0	20	3.01(1.96)	33	3.93(3.50)	0.22	21	3.43(2.83)	32	3.58(2.42)	0.84
4.0	21	2.22(1.67)	32	3.17(4.54)	0.29	20	2.60(1.74)	32	2.41(1.94)	0.72
6.0	16	1.12(1.41)	28	1.95(4.45)	0.37	17	1.09(0.88)	30	0.88(0.96)	0.47
*C*_max _(μg/ml)	21	4.91(2.03)	33	6.92(5.88)	0.08	21	5.67(3.30)	33	6.26(3.41)	0.53
*T*_max _(hour)	21	1.80(0.87)	33	1.67(0.93)	0.62	21	2.17(1.30)	33	1.71(0.88)	0.17
AUC_0–6 _(μg/hour/ml)	21	14.88(7.43)	32	18.07(12.52)	0.25	21	16.52(8.84)	33	17.94(10.36)	0.59

^1^The 2-hour plasma concentrations are calculated as [(2/3)(1.5 hour value+(1/3)(3.0 hour value)].

*C*_max _= observed maximum value for each individual; *T*_max _= the time at which *C*_max _was recorded.

AUC, area under the curve.

The mean calculated 2-hour RMP concentrations of the HIV-infected and HIV-uninfected children on enrolment were 3.90 and 4.78 μg/ml, respectively; although these mean values did not differ significantly (*P *= 0.20), the SD of the HIV-infected group (3.25) was significantly greater (*P *= 0.002) than that of the HIV-uninfected group (1.67). At the 4-month assessment the mean calculated 2-hour RMP plasma concentrations of the HIV-infected and HIV-uninfected groups were 3.96 μg/ml (SD 2.04) and 4.61 μg/ml (SD 2.43), respectively, and neither the means (*P *= 0.33) nor the SD (*P *= 0.40) differed significantly. At the first pharmacokinetic evaluation on enrolment only five (9%) children had calculated 2-hour concentrations greater than 8 μg/ml, while 25 (47%) had values less than 4 μg/ml; although more HIV-infected than HIV-uninfected children had such low values (57% and 41%, respectively), the difference was not significant (*P *= 0.37). At 4 months after treatment commencement three children (6%) had 2-hour RMP concentrations greater than 8 μg/ml and 25 (43%) values less than 4 μg/ml; the proportions of HIV-infected and uninfected children with such very low values were 39% and 43%, respectively (*P *= 0.83). There was no relationship between HIV staging and RMP plasma concentrations either on enrolment or after 4 months of treatment.

In children with and without abdominal nodes visible on ultrasound examination on enrolment the mean *C*_max _concentrations were 5.3 μg/ml (SD 5.2) and 6.3 μg/ml (SD 3.4), respectively, and at 4 months after commencement of treatment 5.3 μg/ml (SD 2.9) and 6.2 μg/ml (3.5), and neither of these differences were significant.

The study was not intended to compare the outcome of treatment, but on discharge from BHCD all children who had a positive culture of *M. tuberculosis *on commencement of treatment were culture-negative. We are aware of one HIV-infected child who has subsequently relapsed, but the children were not specifically followed up to detect relapse. Of the 21 HIV-infected children three (14.3%) had not gained in weight compared to three (8.8%) of the 34 HIV-uninfected children. Sixteen HIV-infected children had an abnormal chest radiograph on study commencement and by the time of discharge six children (37.5%) showed some clearing, five (31.3%) had cleared, but five (31.3%) remained unchanged. Amongst the 34 HIV-uninfected children, 27 initially had chest radiographic abnormalities and of these 15 (55.6%) had improved and 12 (44.4%) had cleared. No association was shown between these features and any aspect of RMP pharmacokinetics.

## Discussion

This study has documented very low serum RMP concentrations in children, of which the great majority received the often recommended standard dosage of 8 to 12 mg/kg body weight. Although there was a trend for lower concentrations in HIV-infected children on the first evaluation 1 month after commencing treatment this was not significant and the 4-month evaluation provided no evidence of differences between the HIV-infected and HIV-uninfected children. Early studies of RMP pharmacokinetics in children under 18 months of age, conducted amongst children not established on RMP, reported an elimination rate similar to that in adults, but a volume of distribution twice that of adults; RMP absorption was noted to be 'erratic and unpredictable' [17]. In comparison to adults young children receiving a RMP dosage of 10 mg/kg appeared to reach considerably lower concentrations of RMP [18]. The relatively low plasma concentrations of RMP reached by children were also evident in other studies and amongst children not established on RMP and receiving a dosage of 10 to 12 mg/kg peak concentrations of 3 to 9 μg/ml were found [18-22]; it should be noted that the highest values derive from a study during which RMP was administered in a suspension [21], peak concentrations amongst children established on RMP ranged from approximately 3 to 5 μg/ml (see [23-25]). On the basis of their findings Hussels et al. [20] considered that children under 6 years of age should receive a RMP dose of 15 mg/kg body weight and older children 10 mg/kg.

Comparison of our results with those from adult studies is difficult due to considerable variation in how pharmacokinetic data are presented and in what detail; thus the AUC may be presented calculated over 0 to 6, 0 to 12 or 0 to 8 hours. Considerable experience in healthy volunteers and tuberculosis patients has led to the suggestion that RMP concentrations of less than 8 μg/ml 2 hours after dosing should be regarded as low and values less than 4 μg/ml as very low and frequent reference is made in the literature to these values [5,8,16]. To enable us to compare our results with these values we calculated the 2-hour RMP serum concentrations in our patients; even allowing for the introduction of a degree of inaccuracy it is clear that, both HIV-infected and HIV-uninfected children, the great majority of whom received the often recommended standard RMP dosages of 8 to 12 mg/kg body weight [26,27], have very low RMP serum concentrations. Close to half the calculated 2-hour concentrations were less than 4.0 μg/ml. This is true of the calculated 2-hour values at both enrolment and after 4 months of treatment. By 4 months the nutritional condition of many of the HIV-uninfected children had improved significantly, but their RMP plasma concentrations remained low, so that poor nutrition cannot fully explain this finding. Equally, although both groups of children had evidence of an acute inflammatory response on enrolment, as indicated by elevated serum CRP concentrations, by 4 months after treatment initiation, although there was continued evidence of an acute inflammatory response in the HIV-infected children, all except two of the HIV-uninfected children had normal CRP values.

Two previous studies amongst adult African tuberculosis patients from Kenya and Botswana reported low RMP concentrations similar to those found in our children and also no difference between HIV-infected and HIV-uninfected patients [6,8]. However, another study from Cape Town reported low RMP plasma concentrations from 88 adult tuberculosis patients with a median 2-hour plasma concentration of 4.4 μg/ml, and found HIV infection to be associated with a 39% reduction in RMP concentrations with 22% of 2-hour values less than 4 μg/ml (see [9]).

In other fields of medicine, such as oncology, it is common practice to determine the dose of an agent for children by reference to body surface area or by making use of percentage of the adult dose as determined, directly or indirectly, from body surface area [28]. Alternatively weight-based bands can be constructed that provide a satisfactory approximation to the use of body surface area and such an approach has been suggested for certain antiretroviral drugs to be administered under programme conditions [29]. Nonetheless recommendations for the dose of RMP for children by the World Health Organization [26,27] and several other bodies make use of the same milligram per kilogram body weight dosage as is recommended for adults. Using a percentage of the adult dose reckoned according to an approximation of body surface area [28] in an hypothetical 10 kg child leads to a RMP dose of 162 mg; if this is then reconverted to a milligram per kilogram dose this would be 16 mg/kg compared with a RMP dosage of 100 mg calculated according to a flat-rate of 10 mg/kg; it is of interest that the dosage of RMP recommended for use in children in the USA is 15 mg/kg with a range of 10 to 20 mg/kg body weight [30].

Do the differences in exposure to lower serum concentrations of RMP (and other antituberculosis agents) that arise from using the same milligram per kilogram body weight dose in children as in adults matter? On the one hand there are a number of studies documenting a satisfactory response of children with tuberculosis to regimens of drugs used in the currently recommended dosages and several recent studies have suggested a lack of any relationship between RMP serum concentrations and sputum culture-conversion in adults [31,32]. On the other hand, it is also becoming apparent that, in the era of HIV/acquired immune deficiency syndrome, any reduction in the dose of antituberculosis agents reflected by frequency and length of treatment, particularly in more severe forms of pulmonary tuberculosis may be associated with an increased risk of relapse [33-36]. In immunocompetent patients a RMP 600 mg dosage, associated with INH, led to more rapid sputum sterilisation of adult pulmonary tuberculosis patients than a dosage of 450 mg (see [37]); Buniva et al. [38], after extensive experience with the development of RMP, stated that *T*_max _was usually reached at between 1.5 and 2 hours in human volunteers and that the mean *C*_max _in 95 individuals after dosages of 600 mg was 12.7 μg/ml (± 0.4), with individual values ranging from 7.6 to 25.9 μg/ml (see [38]). This suggests that the proposed lower limit of normal 2-hour, post-dose concentrations of RMP is appropriate [16]. In studies of early bactericidal activity a RMP dosage of 600 mg was more efficient at killing the metabolically active organisms present in sputum than a dosage of 300 mg, while a dosage of 150 mg had no detectable effect [39,40]. The mean RMP peak serum concentration associated with a dosage of 600 mg in early bactericidal activity studies amongst Chinese patients [39] was 9.53 μg/ml on the second day of treatment and amongst African patients 13 μg/ml and 9.5 μg/ml on the first and fifth days of treatment, respectively [40]. At the less efficacious dosage of 300 mg the mean peak RMP concentration amongst Chinese patients on the second treatment day was 3.27 μg/ml, and amongst African patients, 3.19 and 2.9 μg/ml and on the first and fifth treatment days, respectively.

## Conclusion

This study has documented very low serum RMP concentrations in children, the great majority of whom received the often recommended standard dosage of 8 to 12 mg/kg body weight. While this may be of no consequence in the management of less serious forms of childhood tuberculosis, it might well be very relevant in more severe forms of tuberculosis such as are increasingly encountered in the developing world, especially in association with HIV-infection. From our data we believe that the recommended RMP dose of 10 to 20 mg/kg for children by the American Academy of Pediatrics is a more appropriate dosage range [41]. Studies of higher dosages of RMP in younger children are urgently needed to place the dosing of antituberculosis drugs in children on a more rational foundation.

## Abbreviations

ALT: alanine transferase; AUC: area under the curve; BHCD: Brooklyn Hospital for Chest Diseases; CRP: C-reactive protein; FDC: fixed-dose combination; HIV: human immunodeficiency virus; INH: isoniazid; RMP: rifampin; SD: standard deviation.

## Competing interests

The authors declare that they have no competing interests.

## Authors' contributions

PRD, HSS, DL, GDH and HM collaborated in the writing of the study protocol, KC and DL undertook the nutritional monitoring of the patients, HSS, MW, HM and PS coordinated the clinical and laboratory aspects of the study, JSM undertook the statistical analysis and PRD, HSS, DL and HM were responsible for the writing of the manuscript.

## Pre-publication history

The pre-publication history for this paper can be accessed here:


